# Supplementary material for: High-Sensitivity Pressure Sensors Based on a Low Elastic Modulus Adhesive
Source: Sensors (Basel). 2022 Apr 30;22(9):3425. doi: 10.3390/s22093425 (PMC9103123; doi:10.3390/s22093425)
Supplement: Supplementary file 1 [file sensors-22-03425-s001.zip › sensors-1688133-supplementary.pdf]

Support Information

1. Testing process of the sensor made by screen printing

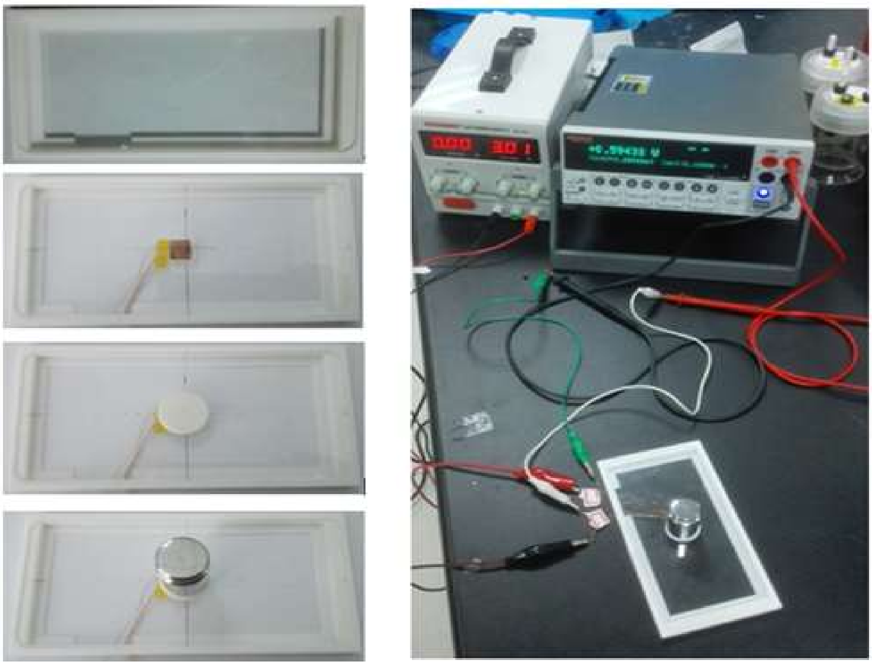

Figure S1. Testing process of the screen-printing sensor.

2. Finite element simulation about the adhesive effect on strain output

2.1 Strain output adopting Wheatstone full bridge and definition of sensitivity

Due to the complexity of the resistance value control of the screen-printing sensor, it is difficult to match the equal bridge arm resistance of the Wheatstone bridge. Therefore, the four resistors on the screen-printing sensor were directly used to form the Wheatstone full bridge, as an analysis of the voltage signal output. Therefore, the difficult problem of matching the equal bridge arm resistance is better solved, and the resistance is basically the same when screen printing with the same printing screen. The initial bridge output signal is close to zero, and the Wheatstone full bridge circuit is also a classic sensor circuit schematic diagram, which is often used to measure the signal output of the sensor.

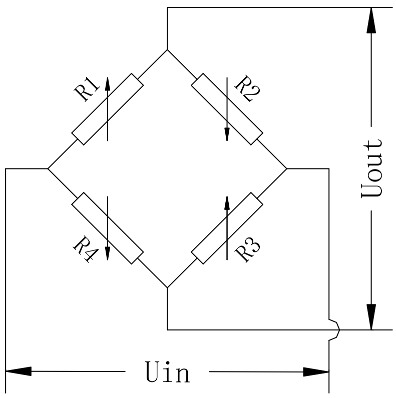

Figure S2. DC Wheatstone bridges.

According to Kirschhoffmann's law in circuitry, as shown in Figure S2, the bridge voltage output：

$U_{out} = U_{in}\frac{R_1R_3-R_2R_4}{(R_1+R_2)(R_3+R_4)}$ (S1)

Let the four arms of the bridge be connected to the four resistors on the front and back of the sensor, when deformed by the force, the change in their resistance values are: $R_1+\Delta R_1$、$R_2+\Delta R_2$、$R_3+\Delta R_3$、$R_4+\Delta R_4$. The output voltage of the bridge at this point is

$U_{out} = U_{in}\frac{(R_1+\Delta R_1)(R_3+\Delta R_3)-(R_2+\Delta R_2)(R_4+\Delta R_4)}{(R_1+\Delta R_1+R_2+\Delta R_2)(R_3+\Delta R_3+R_4+\Delta R_4)}$ (S2)

Since ΔR_i is much smaller than R_i, neglecting ΔR_i in the denominator and the higher term of ΔR_i in the numerator, the voltage output of the bridge can be obtained after sorting and simplifying as

$U_{out} = U_{in}\frac{R_1R_2}{(R_1+R_2)^2}\left(\frac{\Delta R_1}{R_1}-\frac{\Delta R_2}{R_2}+\frac{\Delta R_3}{R_3}-\frac{\Delta R_4}{R_4}\right)$ (S3)

When all four bridge arms have equal resistance values i.e.: $R_1 = R_2 = R_3 = R_4$, and they all have the same sensitivity coefficient, then the relationship $\Delta R_i/R_i = K\varepsilon_i$ is brought into the above equation and we have the output voltage of the bridge as

$U_{out} = \frac{U_{in}}{4}\left(\frac{\Delta R_1}{R_1}-\frac{\Delta R_2}{R_2}+\frac{\Delta R_3}{R_3}-\frac{\Delta R_4}{R_4}\right) = \frac{U_{in}}{4}\cdot K(\varepsilon_1-\varepsilon_2+\varepsilon_3-\varepsilon_4)$ (S4)

The above equation shows a relational combination of the bridge output voltage being proportional to the four strains sensed by the four sensitive grids. Whereas, the sensitivity defined in this thesis is

$\frac{U_{out}}{U_{in}} = \frac{1}{4}\cdot K(\varepsilon_1-\varepsilon_2+\varepsilon_3-\varepsilon_4)$ (S5)

From the above equation, it is known that it is a parameter related only to the sensitivity factor K and the strain ε.

2.2 Finite element model and material selection

The process of attaching the sensor to the glass screen with adhesive is a very complex process involving positioning, pressing, and curing, and it is impossible to assess how the material changes during these processes. In this paper, the finite element model is simplified and the structure of the finite element model is shown in the figure below. The sensitive grid on the sensor is sensing the stress–strain distribution at the location of the PI substrate, so to simplify the model, the sensitive grid and the cover layer are omitted from the simulation and only the stress–strain distribution at the sensitive grid on the PI substrate is concerned. The dimensions of the selected material model are 152.3*72.5*0.7 mm for the glass screen, 8.8*8.3*0.25 mm for the sensor, 4 mm for the middle sensitive grid, two sensitive grids on one side, and two sensitive grids on both sides, one sensor with four sensitive grids, the two sensitive grids on the same side are 0.55 mm and 1.15 mm from the boundary. The sensor is attached to the centre of the glass screen, and the two sensitive grids are located at the centre of the loading; for the loading and boundary, the glass screen is loaded at the centre, loaded with 100g weight, and the glass screen is bounded around; the material parameters, in order to simulate the influence of adhesives with different elastic modulus on the strain output in a wider range, the adhesive used in this paper is chosen for the influence of the elastic modulus of the adhesive on the strain output The modulus of elasticity is E = 0.01 MPa to 5000 MPa and u = 0.2 to 0.4, which are specified in the table.

Table S1. Material performance parameters.

Parameters \ Materials	Glass	Polyimide films	3M Double sided adhesive	Loctite 401 adhesive	Adhesive variables
Modulus of elasticity /MPa	74000	6000	0.4	2500	0.05~5000
Poisson's ratio	0.22	0.34	0.4	0.3	0.4
Density /kg/mm^3	2.5*10^{-6}	1.4*10^{-6}			1*10^{-6}

It is assumed that the materials used are all isotropic and that for isotropic materials the following equation exists for the shear and elastic moduli, G=E/[2(1+v)], G is the shear modulus

and E is the elastic modulus, both units in MPa, and μ is the Poisson's ratio.

Table S2. Measurements of the modulus of elasticity of 3M double sided tape on the tensile tester.

Force (gf)	Tensile strength (kPa)	**modulus of elasticity** (kPa)	Deformation at the point of maximum force (mm)	Elongation at break (%)	Higher Yield strength (kPa)	Lower yield strength (kPa)
68.49	516.64	400.85	20.22	183.7	287.51	272.32

DG-Path01 and DG-Path02 simulate the strain distribution on the two sensitive grid paths on the PI backing surface of the 3M double-sided adhesive; DG-Path03 and DG-Path04 simulate the strain distribution on the two sensitive grid paths on the PI backing surface of 3M double-sided adhesive; HY-Path01 and HY-Path02 simulate the strain distribution on the two sensitive grid paths on the PI backing surface of Loctite 401 adhesive. HY-Path01, HY-Path02 are strain distributions on the two sensitive grid paths on the PI adhesive surface with Loctite 401 adhesive; HY-Path03, HY-Path04 are strain distributions on the two sensitive grid paths on the PI adhesive surface with Loctite 401 adhesive

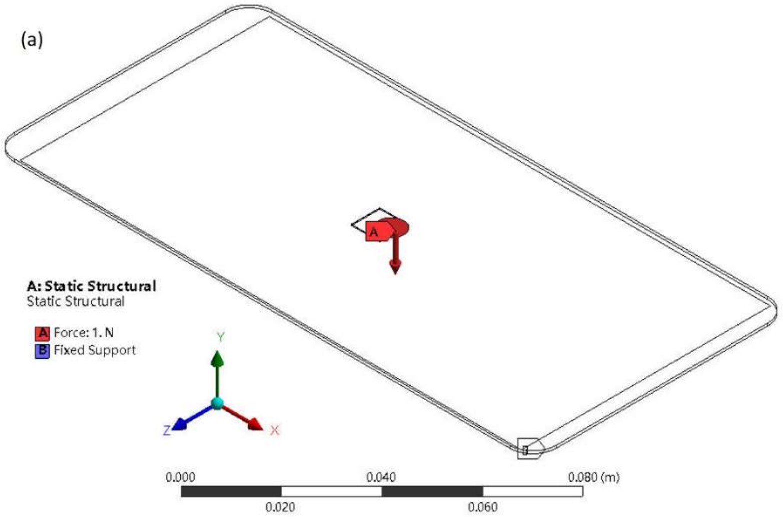

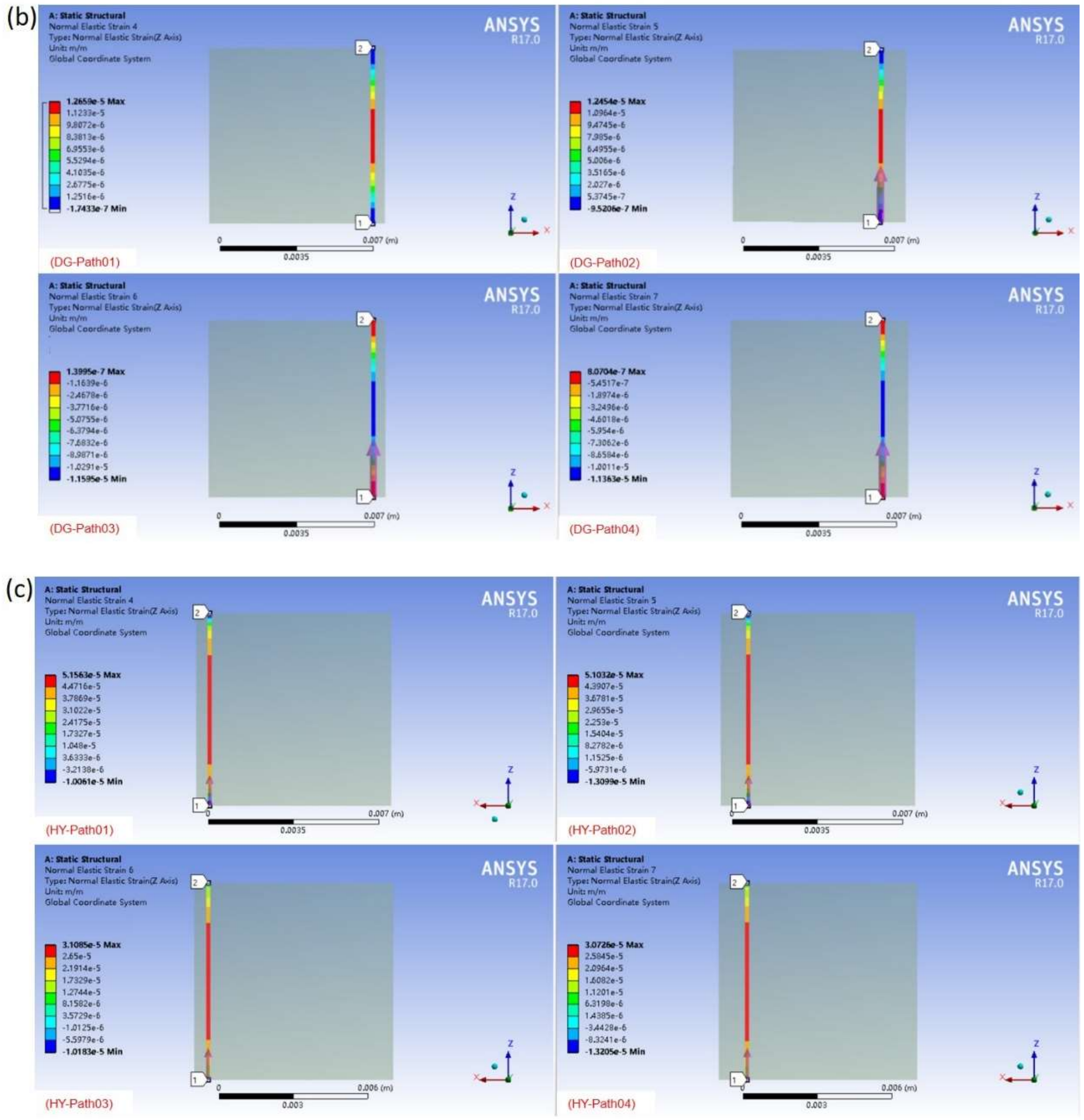

Figure S3. (a) Assembly relationship model with loading and boundary schematics; (b) Results of 3M double-sided adhesive paste sensor (M3) simulation, DG-Path01 for M3 first strip, DG-Path02 for M3 second strip, DG-Path03 for M3 third strip, and DG-Path04 for M3 fourth strip, corresponding to the strain distribution in the length of the sensitive grid; (c) Results of Loctite 401 adhesive simulation results, HY-Path01 for L4 first strip, HY-Path02 for L4 second strip, HY-Path03 for L4 third strip, and HY-Path04 for L4 fourth strip, corresponding to the strain distribution in the length of the sensitive grid.

Figure S3b shows the strain contour figures of the 3M double-sided adhesive paste sensor (M3) simulation. DG-Path01 represents M3 first sensitive grid, and DG-Path02 represents M3 second sensitive grid, and they are on the backing surface (Non-Glue Surface) on the z-axis direction; Red in the middle represents large positive strain in the middle, and blue on both sides represents small negative stain on both sides.DG-Path03 represents M3 third sensitive grid, and DG-Path04 represents M3 fourth sensitive grid, and they are on the adhesive surface (Glue Surface) on the z-axis

direction. Blue in the middle represents a large negative strain in the middle, and red on both sides represents a small positive stain on both sides. Figure S3c shows the strain contour figures of the Loctite 401 adhesive paste sensor (L4) simulation. HY-Path01 represents L4 first sensitive grid, and HY-Path02 represents L4 second sensitive grid, and they are on the backing surface (Non-Glue Surface) on the z-axis direction. Red in the middle represents a large positive strain in the middle, and blue on both sides represents a small negative stain on both sides. HY-Path03 represents the L4 third sensitive grid, and HY-Path04 represents L4 fourth sensitive grid, and they are on the adhesive surface (Glue Surface) in the z-axis direction. The changed trend of HY-Path01 and HY-Path02 are similar to that of HY-Path01 and HY-Path02. Red in the middle represents a large positive strain in the middle, and blue on both sides represents a small negative stain on both sides. For a better comparison, Table 1 gives the comparative total strain output and sensitivity for the simulations and experimental test of M3 and L4, respectively.

Table S3. Different compressive and tensile strains developed on the PI adhesive side and PI backing side for adhesives with different elastic moduli.

Adhesive Layer / Description	Soft adhesive<br>Modulus of elasticity<10MPa	Hard adhesive<br>Modulus of elasticity≥10MPa
Strain near the adhesive surface	Negative strain	Positive strain
Strain away from the adhesive surface	Positive strain	Positive strain
Adhesive material	3M double-side adhesive	Loctite adhesive
Shear modulus	Low	High

Measurement of double-sided adhesive thickness

Experimental process: Using a spiral micrometer, the overall thickness of the double-sided adhesive was measured first, then the double-sided adhesive was torn apart and the thickness of the release paper and the thickness of the substrate were measured separately. Finally, the overall thickness was subtracted from the thickness of the release paper and the substrate to obtain the thickness of the double-sided adhesive, and the thickness of the adhesive layer of the double-sided adhesive was approximately 0.056 mm according to the above measurement and calculation method.

Table S4. Thickness of the 3M double-coated adhesive layers.

**Samples / Description**	**1#**	**2#**	**3#**	**4#**	**Average value**
**Overall thickness of double-sided adhesive** (mm)	0.262	0.259	0.262	0.264	
**Thickness of release paper** (mm)	0.102	0.099	0.102	0.103	
**Thickness of substrate** (mm)	0.104	0.103	0.105	0.105	
**Adhesive thickness**(mm)	0.056	0.057	0.055	0.056	0.056
**Sample width** (mm)	10	10	10	10	
**Sample length** (mm)	40	40	40	40	

Testing the modulus of elasticity of double-sided adhesive

Experimental process: In order to obtain the relevant parameters of the modulus of elasticity for the finite element simulation, tensile experiments were carried out on the adhesive layer of the double-sided adhesive. 40 mm x 10 mm sections of double-sided adhesive were taken, as shown above, and the tensile experiments were carried out on a tensile testing machine.

Table S5 Data of the tensile test results

Samples	Maximum force/(gf)	Tensile strength /(kPa)	Modulus of elasticity /(kPa)	Deflection at max-force /(mm)	Elongation at break /(%)	Higher yield strength /(kPa)	Lower yield strength /(kPa)
**11#**	75	636.77	362.2	23.91	264.06	350.99	316.51
**22#**	68.49	516.64	400.85	20.22	183.714	287.51	272.32
**33#**	73.98	821.2	322.27	35.37	290.914	371.02	370.2
**44#**	43.23	756.97	462.61	26.35	266.486	340.62	344.48
**55#**	53.96	944.98	508.9	13.83	161.843	423.79	438.32

The above table shows that the modulus of elasticity of the double-sided adhesive is approximately 0.4 MPa.

Measurement of Loctite 401 instant adhesive thickness

Experimental scheme: first use the spiral micrometer to measure the thickness of the mobile phone glass screen, then cut the sensor size of the PI sample, using the spiral micrometer to measure the corresponding thickness of each sample, and using Loctite 401 instant adhesive to paste the PI sample to the mobile phone glass screen, with the PTFE sheet pressed for about 1 minute, placed a day later to measure the overall thickness. Finally, the overall thickness minus the PI sample thickness and the thickness of the mobile phone glass screen were calculated. The final thickness of the adhesive layer is obtained by subtracting the thickness of the PI sample and the thickness of the mobile phone glass screen.

Table S6. Thickness of the adhesive layer of the Loctite 401 instant adhesive (unit：mm).

Samples / Layers	21#	22#	23#	24#	25#	26#	27#	28#	29#	Average value
**Glass Screen Thickness**	0.7195	0.7195	0.7195	0.7195	0.7195	0.72	0.72	0.72	0.72	
**PI Thickness**	0.252	0.244	0.246	0.249	0.249	0.244	0.245	0.248	0.244	
**Total thickness**	1.003	0.994	0.998	0.995	1.000	0.998	0.994	0.997	0.991	
**Thickness of Loctite 401 Adhesive**	0.0315	0.0305	0.0325	0.0265	0.0315	0.034	0.029	0.029	0.027	0.0302

Thickness of Loctite 401 adhesive = Total thickness – Glass screen thickness – PI thickness

Through the above measurement results, the adhesive layer thickness of the Loctite 401 after curing is 0.03 mm. The modulus of elasticity of the Loctite is measured with reference to its technical specifications

Response Time: Because the connection between the sensor and the tested component is a kind of soft adhesive layer, which necks after being subjected to force and transfers the force to the sensor, the effect of soft adhesive bonding on the dynamic response time (about 36 ms, Figure S4) of the sensor is worse than that of hard adhesive bonding theoretically.

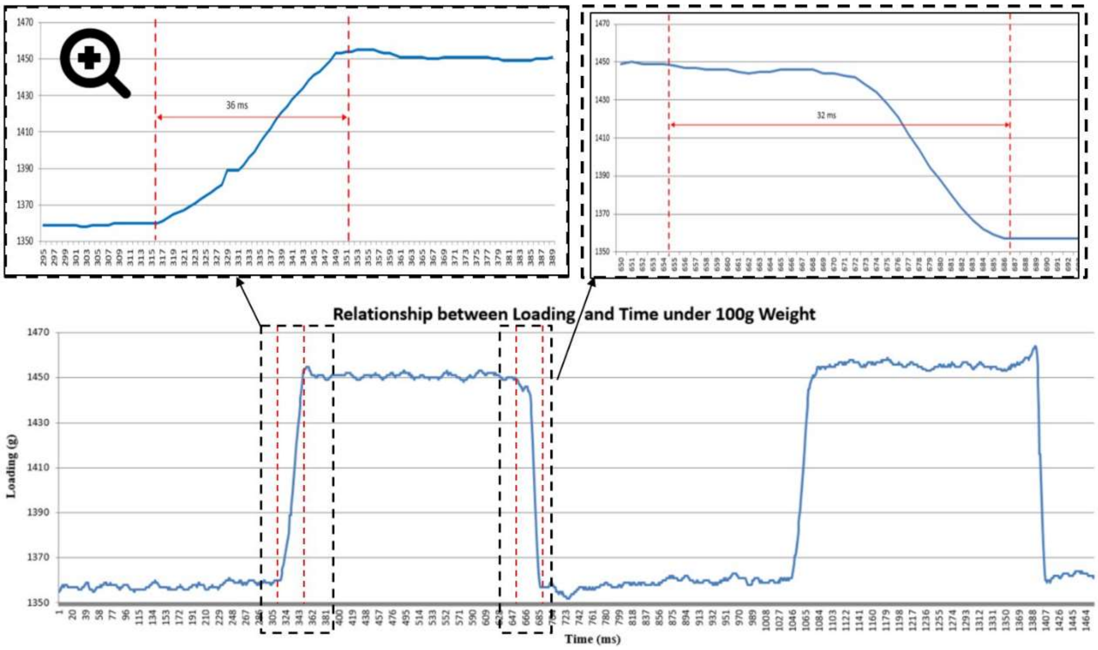

Figure S4. Loading and Response Time of this Study.
